# Parents’ self-reporting of Child Physical Maltreatment (CPM) in a low-middle-income country

**DOI:** 10.1186/s12888-023-04947-x

**Published:** 2023-07-13

**Authors:** Mai SeifElDin Abdeen, Mostafa Ahmad Hashim, Marwa Mohamed Ghanem, Nouran Yousef Salah El-Din, Zeinab Mohamed El Nagar

**Affiliations:** 1 Psychiatry Department, Faculty of Medicine, Ain Shams University, 38 Abbaseya St, Cairo, Egypt; 2 Psychiatry Department, Faculty of Medicine, Helwan University, Cairo, Egypt; 3 Pediatrics Department, Faculty of Medicine, Ain Shams University, Cairo, Egypt

**Keywords:** Child maltreatment, Parents’ stress, Depression, Anxiety, COVID-19

## Abstract

**Background:**

Because of COVID-19 pandemic, families across the world are experiencing new stressors that threaten their health, and economic well-being. Such a stress may jeopardize parents-children relationship. We aim to investigate the magnitude of child physical maltreatment (CPM) by parents in Egypt during the COVID-19 pandemic, to relate it to parents’ stress, and to identify other potential risk factors.

**Methods:**

This cross-sectional study assessed parent-reported CPM and their personal experience of stress, depression, and anxiety among a sample of Egyptian parents using an electronic survey. It included sociodemographic data, Depression, Anxiety and Stress Scale (DASS-21), and the Child physical maltreatment scale (CPMS). We also briefly assessed COVID-19 -related data.

**Results:**

Out of 404 respondents, (62.9%) and (32.9%) reported performing minor and severe CPM toward their children during the past 3 months, respectively. The age of youngest child, and anxiety score were significantly correlated with both minor and severe forms of CPM. While number of children, and online education system ratings were only significantly correlated with severe CPM. Parental definition of CPM was significantly correlated to minor CPM, but not to severe CPM.

**Conclusions:**

CPM by parents is not uncommon in Egypt, especially during the COVID-19 pandemic. These findings highlight the importance of regular support and intervention that help parents learn parenting skills and the use of non-violent child disciplining methods.

**Supplementary Information:**

The online version contains supplementary material available at 10.1186/s12888-023-04947-x.

## Introduction

The COVID-19 pandemic poses new challenges to every family across the world, given the novelty and uncertainty concerning this disease [11]. Variable models of prevention have been adopted in different countries in response to the COVID-19 pandemic. Preventive measures in Egypt included suspension of education (from March 15^th^ until October 16^th^, 2020), and closure of Mosques and churches. There was partial lockdown with “stay-at-home” orders [9, 22].

Such a global crisis and its percussions, including increased economic stress, unemployment, lockdown, social isolation, physical and mental health concerns, and the shift to home-schooling, may contribute to increased stress among parents [21, 24, 39]. When feeling overwhelmed by the crisis, and when individuals’ resources are insufficient to cope with stressors, parents experience high levels of stress. They may vent these negative emotions through improper behaviours, such as physical or verbal abuse, particularly in families with history of domestic violence or child maltreatment [17, 43]. For example, parents having burnout reported more conflicts with their partners and higher levels of child abuse and neglect [31]. An interventional research even demonstrated that child abuse and neglect could be reduced by targeting levels of parental stress [10].

Amid the COVID-19 crises, increasing reports of child physical maltreatment (CPM) during periods of quarantine and lockdowns emerged [17]. In contrast to these reports, rates of child maltreatment *officially* reported decreased during the pandemic, which could be attributed to social isolation, and reduced in-person contact with teachers, doctors, child protective services and other professionals, who mainly report such incidents [13, 24]. Despite the underestimated number of maltreatment cases, public health organizations emphasized the increased risk for child maltreatment, especially among families that were abusive prior to the pandemic [39, 43].

Unfortunately, limited studies tackling CPM have been done in Egypt, due to the lack of population-based statistics, and unreliable official records [4]. Researchers often resort to parent or child reports to measure CPM incidence, despite evidence that parents usually underreport maltreatment of their children [34]. Still, it provides a better estimate of CPM occurrence,up to 70 times higher than the estimates derived from official reports [40].

Hard as it is, such a problem is also challenging to study in the Arab countries, and in Egypt, due to the cultural reluctance to report CPM [6]. In the Arab world, research about CPM is scarce, and available ones show it is common and underreported [4].

El-Defrawi, Atef, Ragab & Sobhy [18] study (done on a total of 672 Egyptian parents) reported that they tend to use force in the form of beating to discipline children, particularly when the child shows disruptive behaviour or delinquency. And that such practices increase in severity, and it may even be fatal in low socio-economic families, with lower educational level of parents.

To date, very few studies addressed CPM and parents’ stress in low & middle-income countries [11, 13, 32]. The current study aims, firstly, to estimate the magnitude of CPM in an Egyptian convenient sample during the COVID-19 pandemic. Secondly, to investigate the association between CPM and parental psycho-social stress (stress, anxiety, depression). We hypothesize a robust relation between CPM to parents’ stress. This study can help generate a base of knowledge for monitoring and evaluating CPM and family violence in low & middle-income countries.

## Methods

A cross-sectional research design was employed in this study. The study group comprised a total of 404 parents recruited (using convenient sampling) by disseminating the electronic survey link through social media (Facebook groups of parents of different school types). The recruitment statement specified that respondents should be Egyptian parents living in Egypt; there were no listed exclusion criteria.

Due to the lack of awareness of CPM, the social stigma attached to CPM in the Egyptian culture, and the possible unease of participants to report such incidents [4], we resorted to conduct this study using an anonymous online survey. The online survey was open for completion from January 2021 to March 2021. Completion of the survey was entirely voluntary and anonymous. The study protocol was approved by the Ain Shams university ethical committee with the number FMASU R 128/2021. An informed consent statement was obtained from participants.

### Assessment

Demographics of the of 404 participants’ were assessed, including age, gender, education level, marital status, perceived family income(high/average/low), residence, and number and age of children. COVID-19 stressors were briefly assessed using investigator developed questions, for fear of being a potential confounding factor. Parents were asked if they tested positive for COVID-19 or knew someone who had COVID-19 infection. Using a scale from (0–10), parents were asked to indicate their level of worry of the COVID-19 pandemic, and to rate how stressful was the social isolation at home, and the shift to online education system.

Moreover, parents were asked one question*,* if they-*themselves*- were subjected to physical maltreatment as children (never/ rarely/ sometimes/ often/ very often).

Participants completed 2 sets of scales, namely, the Depression, Anxiety and Stress Scales (DASS-21) [27] and the Child physical maltreatment scale (CPMS) [28].

*The DASS-21 tool* consists of 3 self-report scales that assess depression, anxiety, and stress during the past 7 days on a four-point scale between zero and three with higher scores indicating severity. Scores of statements under the same emotional category (seven statements each) were summed and multiplied in two. Out of 42, the cut-off values for mild to moderate depression, anxiety, and stress were 10, 8, and 15, and for severe to very severe were 21, 15, and 26, respectively [12, 27]. The Cronbach’s α of the DASS-21 scale in the current sample was 0.939. the arabic version of DASS-21 tool was used [5].

*Child physical maltreatment (CPM)* was evaluated using the CPM scale [28].

CPM was assessed based on items used in various published studies (e.g., [7, 14, 37, 42]. The scale included 7 items:

- Pinched, shook, pushed or shoved a child
- Hit child’s hand, back, arm or leg with hand
- Hit child’s buttocks with hand
- Hit child’s buttocks with an object
- Hit child’s face or head with hand
- Kicked a child with a foot or hit with a fist
- Hit elsewhere (not buttocks) with an object

The first three items are defined as minor CPM. The last four items are classified as severe CPM [28]. The Cronbach’s α of the 7-item scale in the current sample was 0.743.

#### Frequency of CPM

The scale asked parents how often they performed the behaviours in these items towards their children during the past 3 months. The response categories included never, 1–2 times, 3–5 times, 6–10 times and > 10 times. The midpoints of response categories of the scale were used to define the frequency scores during the past 3 months, that is, “0” for “never,” “1.5” for “1–2 times,” “4” for “3–5 times,” “8” for “6–10 times,” and “15” for “more than 10 times.” [37].

#### Respondents’ definition of CPM

After each item, the respondent was asked whether he/she believed that the behaviour was an abusive behaviour. Responses were categorized by a 3-point measure, on which “1” indicated “yes,” “2” indicated “unsure,” and “3” indicated “no.” Higher scores reflected low awareness of CPM.

### Statistical analysis

Statistical analysis was done on a personal computer using IBM© SPSS© Statistics version 26 (IBM© Corp., Armonk, NY).

Data were described in the form of number and percentage, and mean SD. Correlations among numerical variables were tested using Pearson correlation test (r). *P*-values ≤ 0.05 were considered statistically significant.

Multiple linear regression analysis was used to assess predictors of the frequency of different forms of CPM. The confidence interval was set to 95% and the margin of error accepted was set to 5%. So, the *p*-value was considered significant at the level of < 0.05.

## Results

### Demographic and clinical characteristics of the sample

This is a cross sectional study of Egyptian parents reporting Child Physical Maltreatment (CPM). A total of 404 participants were enrolled in study (377 mothers and 27 fathers), with mean age of (37.4 ± 5.7) years. Most participants reported middle family income (73.5%), and almost all lived in urban location. Participants were mostly married and had a higher education degree. Almost third of parents reported being abused physically themselves as children (Table1).

**Table 1:** Sociodemographic characteristics, the DASS-21 scale and COVID-19-related variables of the sample

		**Total** *N* = 404 (%)
**Sociodemographic characteristics**		
**Age of parent** (Mean ± SD)		37.4 ± 5.7
**Gender**	Mothers	377 (93.3%)
	Fathers	27 (6.7%)
**Marital Status**	Married	380 (94.1%)
	Divorced/ widows	24 (5.9%)
**Education**	High university degree	399 (98.8%)
	Middle/high school	5 (1.2%)
**Family Income**	Low	3 (0.7%)
	Middle	297 (73.5%)
	High	104 (25.7%)
**Residence**	Urban	395 (97.8%)
	Rural	9 (2.2%)
**Number of Children** Median (range)		2 (1 – 5)
**Age of youngest child** Median (IQR)		5 (3 – 9)
**Parent PH**^a^** of being physically abused themselves as children**	Never	170 (42.1%)
	Rarely	90 (22.3%)
	Sometimes	123 (30.4%)
	Most of the time	21 (5.2%)
COVID-19 related data		
PH of positive COVID-19 test		85 (21%)
Someone close to you tested positive for COVID-19		202 (50%)
COVID-19 worry scale (0–10) (where 0 is the worst and 10 is the best) (mode)		8
Social isolation scale (0–10) (where 10 is the worst and 0 is the best) (mode)		7
School online education rating (0–10) (where 0 is the worst and 10 is the best) (mode)		5
Minor CPM frequency (Mean ± SD)	4.2 ± 6.6	254 (62.9%)
Severe CPM frequency (Mean ± SD)	1.6 ± 4.2	133 (32.9%)
DASS-21 scale		
Stress Score (Mean ± SD)		21.2 ± 9.9
Anxiety Score (Mean ± SD)		16.1 ± 8.7
Depression Score (Mean ± SD)		17.1 ± 9.5

*SD* Standard deviation

^a^*PH* Past history

^b^*IQR* Interquartile range

**Fig. 1:**
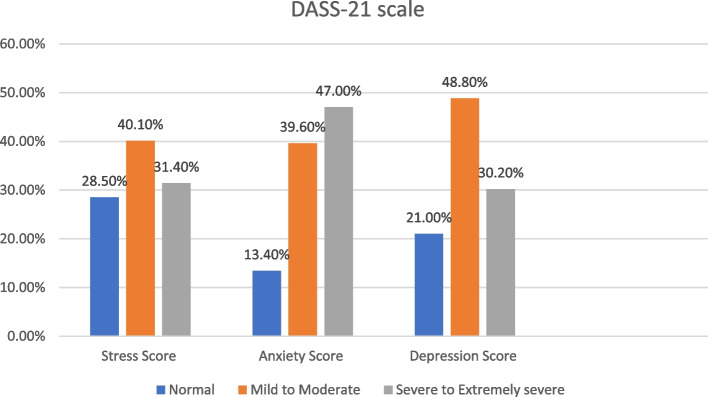
The degree of stress, anxiety, and depression in participants, using DASS-21

According to DASS-21 cut-off scores, participants’ responses were divided to 3 degrees; normal, mild to moderate, and severe to very severe. According to participants’ responses, 40.1%, 39.6% and 48.8% showed mild to moderate depression, anxiety, and stress, respectively. 31.4%, 47% and 30.2% of the participants reported severe to extremely severe symptoms of stress, anxiety, and depression, respectively (Fig. 1).

### Participants’ definition, and frequency of CPM

The mean frequencies of occurrence of minor and major CPM in past 3 months were 4.2 ± 6.6 and 1.6 ± 4.2 times, respectively (Table 1). The most common forms of CPM were ‘Hitting a child’s hand, back, arm or leg with hand’ (46.3%), followed by ‘Pinching, shaking, pushing or shoving a child’ (46%) and then ‘Hitting a child elsewhere (not buttocks) with an object’ (15.8%) (supplement table S1). It is important to mention that (22.5%) refused to mention frequency of one or more form of CPM.

As shown in Table 2, the minor CPM definition score (denoting minor CPM definition by parents) was significantly negatively correlated with the frequency of minor CPM in past 3 months. However, parental definition of severe CPM was not significantly correlated with severe CPM frequency.

**Table 2:** Correlation of reported frequency of CPM (in past 3 months) in the studied subjects with parent’s age, age and number of children, COVID 19 and the DASS-21 data

		Minor CPM frequency	Severe CPM frequency
Age of parent	**r**	-0.127	-0.002
	***P*-value**	0.011*	0.976
Age of youngest child	**r**	-0.217	-0.107
	***P*-value**	< 0.001**	0.032*
Number of children	**r**	0.066	0.188
	***P*-value**	0.186	< 0.001**
COVID-19 worry scale (0–10), where 0 is the worst	**r**	0.000	0.026
	***P*-value**	0.998	0.601
Social isolation (1–10), 10 worst	**r**	-0.024	0.052
	***P*-value**	0.637	0.300
Online education (0–10), where 0 is the worst	**r**	-0.044	-0.135
	***P*-value**	0.385	0.007**
Minor CPM definition	**r**	-0.146	-0.037
	***P*-value**	0.003**	0.459
Severe CPM definition	**r**	-0.075	-0.062
	***P*-value**	0.136	0.211
Stress score	**r**	0.185	0.093
	***P*-value**	< 0.001**	0.061
Anxiety score	**r**	0.207	0.154
	***P*-value**	< 0.001**	0.002**
Depression score	**r**	0.173	0.060
	***P*-value**	< 0.001**	0.228

^*^ = statistically significant < 0.05, ** = highly statistically significant < 0.01

**Fig. 2:**
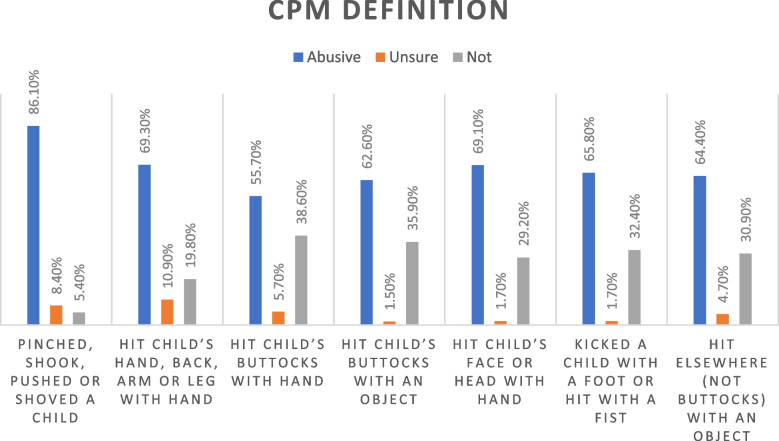
CPM definition according to participants

Participants’ definitions of CPM are demonstrated in Fig. 2. Out of all parents, 5.4%, 19.8% and 38.6% defined the three items of minor CPM (Pinched, shook, pushed or shoved a child- Hit child’s hand, back, arm or leg with hand- Hit child’s buttocks with hand) as *not* abusive, respectively. As for severe CPM, 35.9%, 29.2%, 32.4% and 30.9% viewed the four items (Hit child’s buttocks with an object- Hit child’s face or head with hand-Kicked a child with a foot or hit with a fist-Hit elsewhere (not buttocks) with an object) as *not* abusive, respectively.

Regarding minor CPM, Table 2 shows that the age of parents, age of the youngest child, were negatively correlated with minor CPM frequency. While minor CPM frequency was positively correlated with all three depression, anxiety, and stress scores.

On the other hand, major CPM frequency was negatively correlated with the age of youngest child, number of children, and online education ratings. And it was only positively correlated with the anxiety score.

### Multiple regression analysis

The variables with a significant correlation with frequency of both forms of CPM (in past 3 months) were included in the multiple regression analysis (Table 3). It showed that the age of youngest child, minor CPM definition score, and anxiety score stood out to be of significance as predictors of minor CPM frequency in past 3 months. On the other hand, regarding severe CPM frequency; the age of youngest child, number of children, online school education ratings, and anxiety score remained significant.

**Table 3:** Multiple regression analysis for the significant variables associated with reported

	Unstandardized Coefficients		Standardized Coefficients	t	Sign
	**B**	**SE**	**Beta**		
Dependent Variable: Minor CPM frequency score (in past 3 months)					
(Constant)	5.672	3.090		1.836	0.067
Age of parent	0.007	0.092	0.006	0.072	0.942
Age of youngest child	-0.426	0.119	-0.258	-3.587	< 0.001**
Minor CPM definition score	-0.478	0.196	-0.119	-2.434	0.015*
Stress score	-0.037	0.073	-0.054	-0.502	0.616
Anxiety score	0.196	0.080	0.259	2.447	0.015*
Depression score	-0.019	0.066	-0.027	-0.288	0.773
Dependent Variable: Severe CPM frequency score (in past 3 months)					
(Constant)	-0.157	0.844		-0.186	0.852
Number of children	1.039	0.241	0.207	4.309	< 0.001**
Age of youngest child	-0.103	0.051	-0.098	-2.029	0.043*
School online education (0–10), where 0 is the worst	-0.195	0.077	-0.123	-2.545	0.011**
Anxiety score	0.071	0.023	0.146	3.036	0.003**

*CPM* Child physical maltreatment

^*^ = statistically significant < 0.05, ** = highly statistically significant < 0.01

## Discussion

There is concern that the COVID-19 pandemic may carry hidden threats to children’s safety worldwide [24]. Given the increased contact between family members amid lockdown together with an economic crisis, this may have created the ideal conditions for an increase in CPM [34]. Such a rise will possibly be underestimated given the difficulty to track its incidence during the pandemic.

We found that the parental self-reported CPM (during past 3 months) in the study sample was common, with 62.9% and 32.9% of parents reported minor and severe CPM towards their children, respectively. It is hard to determine if it is because of COVID-19 crises or not, due to the lack of pre-pandemic data. Regarding parental stress, only parents’ anxiety was correlated to both minor and severe CPM frequency in past 3 months, in contrast depression and stress were not of significance after multiple regression analysis. Parental definition of CPM was predictive for minor CPM frequency only, but not severe CPM frequency. In addition, younger age of children was negatively correlated with both minor and severe CPM frequency. While a greater number of children was only positively correlated with severe forms of CPM.

In one of the few Egyptian studies underwent during the pandemic, 90.5% of children were subjected to violent discipline, and 43.2% encountered severe physical punishment [1]. These rates were higher than that in our study, which could be attributed to the use of different tools, and the time of sampling.

To the best of our knowledge, few studies addressed CPM in Low- and Middle-Income Countries or the Arab world. For example, Maker, Shah, and Agha [29] estimated parent–child physical violence to be 78% in a Latina sample, 73% in South Asian and Middle Eastern samples, and 65% in an East Asian sample. Two Chinese studies also reported comparable figures to our results,in Ma et al. [28] parental self-reported CPM was 42.7% for minor CPM and 21.6% for severe CPM. Similarly, Tang [38] showed prevalence rates between 46 and 52.6% for minor CPM. And in agreement with our results, CPM was more frequently inflicted on younger as compared to older children.

However, a recent Iranian study, showed much lower rates that that in our sample, with 27% of elementary school children reported being physical abused at home [33]. And, in contrast to our findings, there was insignificant effect of child’s age on child abuse. This variation may be explained by the different age groups of the sample, or cultural variation.

Our findings were, however, comparable to previous pre-pandemic Egyptian and regional population-based studies, where pre-pandemic studies reported rates of CPM between 55 and 72% in their samples [4, 19, 44]. However, a previous pre-pandemic study of severe CPM in Egypt showed much less rates than ours (7.6%) versus (32.9%) in our sample [2]. This discrepancy may be explained by the different age groups included in the samples, as well as contextual factors.

Though the age of parents was positively correlated with minor CPM frequency, it was not of significance after controlling for other factors in multiple regression analysis. This came in contrast to other studies, associating physical means of punishment with younger age of parents [3, 15]. This is quite understandable, since younger parents may have less parenting abilities, experience, and knowledge to manage their children, and have more stresses due to limited financial resources. However, in agreement with our findings, [35] found no consistency of parental age as a risk factor for maltreatment.

### Respondents’ definition of CPM

In this study, parental definition of CPM significantly impacted the frequency of minor CPM, but not severe CPM. This came in contrast to some studies showing CPM to be associated with parents’ approving of physical discipline [8, 16]. However, one Chinese study had similar results to ours demonstrating that parental definition of CPM had a significant impact on the frequency of minor CPM, but not on severe CPM [28]. Why that was significant for minor, but not severe CPM frequency, warrants further study. However, this finding denotes that frequency of severe CPM mostly occurred regardless of parents’ definition of CPM, i.e., whether parents thought it was an abusive act or not. This aligns with the idea that all parents can become abusive under certain circumstances [21].

In the current sample, parents who regarded items of minor CPM as non-abusive ranged from 5.4% to 38.6%, and those who viewed major CPM as non-abusive ranged from 29.2% to 35.9%. Attitudes toward physical punishment of children as a form of discipline may be affected greatly by unawareness and cultural norms [28]. According to Egyptian traditional culture, some CPM behaviors, especially the minor CPM behaviors, may not be considered abusive, because corporal punishment is somewhat accepted as a disciplining approach [36]. Our results support this observation, given that almost third of the parents in this study reported being abused physically *themselves* as children.

In concordance with our results, a study underwent in Saudi Arabia reported that around 34% of the participants reported a history of CPM [3]. In fact, many researchers have found that parents’s own rearing history contributes to the way they practice parenting [23]. For instance, many child protective services involve parents that have been abused as children. Such early traumatic experiences can adversely impact their caregiving behavior and increase risk of the intergenerational transmission of child abuse [41]. We could not determine whether parental past history of CPM could explain the high rates of CPM in the sample in the current study design. Yet, identifying such high-risk parents and providing them with training programs could be effective in the prevention of CPM.

### CPM and COVID -19 variables

Perceived COVID-19 worry, and social isolation were not related to the frequency of any form of CPM. While online education parents’ ratings had significant correlation with severe CPM. In agreement with our results, Brown et al. [11] found that the cumulative stressors from COVID-19 did not significantly relate to increased risk of CPM potential in their sample. Although stress, in general, is strongly associated with CPM, however stressors specific to COVID-19 may not intensify CPM risk. These results need replication using standardized tools.

### CPM and Parents’ stress (using DASS-21)

Out of all participants, 31.4%, 47% and 30.2% reported severe to extremely severe symptoms of stress, anxiety, and depression, respectively. These results are consistent with prior research showing that during the COVID-19 pandemic nearly a third of adults reported clinically meaningful symptoms of anxiety and depression [11, 25]. In another Italian study, 17% of their sample experienced significant parenting-related exhaustion in COVID-19 pandemic [30]. El‑Zoghby, Soltan, & Salama [20] Egyptian study of COVID-19 impact on mental health, showed that (41.4%) of their sample suffered a severe impact.

*Regarding relation to CPM,* we found that after multiple regression, only parents’ anxiety scores stood out to be of significance in predicting both minor and severe CPM frequency.

In agreement to our results, Brown et al., [11] and Lawson et al. [24] studies demonstrated that parental anxiety was a significant predictor of CPM during the pandemic. One Egyptian study showed that there was a strong association between psychological impact of COVID-19 pandemic on parents and them practicing violence against their children [1].

## Limitations

Although the CPM rates are apparently common in our study, they are mostly minimum estimates of the actual figures. Several important limitations should be borne in mind when interpreting these results. First, the observational nature of the present study and the lack of pre-pandemic data to compare with, make it difficult to draw causal inferences. Moreover, convenience samples lack generalizability. Second, the current study only studied physical maltreatment, we did not assess emotional abuse, neglect, nor sexual abuse. Third, some parents may refrain from reporting CPM behaviors because of shame. Fourth, using an online survey may have caused a sort of selection bias as it may only be restricted to those who had smart devices and internet access. This is displayed in the fact that most participants were of the middle class and very few were from a low socio-economic class. This may have underestimated the gravity of the situation. Finally, self-selection bias may exist if the non-respondents were either too abusive to their children or not at all abusive and therefore not interested in this survey.

Despite limitations, this study has important clinical implications such as;

There are unmet needs by the child welfare system in Egypt even before the COVID-19 pandemic. This could be overcome, by providing multiple points of contact to reach parents and children (e.g., primary health care, public schools, and religious institutions). Finally, parents’ stress is a potentially modifiable risk factor contributing to CPM. Parents’ training programs can be an effective solution [26].

## Supplementary Information

**Additional file 1:** **Table(S1).** CPM 7-items frequency during past 3 months reported by the studied participating parents. **Figures (S1).** Curve estimation ofsignificant predictors of minor and severe CPM frequency (in multipleregression).

## Data Availability

Data will be available upon request from the corresponding author.

## Abbreviations

CPM: Child Physical Maltreatment
DASS-21: Depression, Anxiety and Stress Scales
CPMS: Child physical maltreatment scale
