# Supplementary material for: Parents’ self-reporting of Child Physical Maltreatment (CPM) in a low-middle-income country
Source: BMC Psychiatry. 2023 Jul 13;23:506. doi: 10.1186/s12888-023-04947-x (PMC10339565; doi:10.1186/s12888-023-04947-x)

Table (S1). CPM 7-items frequency during past 3 months reported by the studied participating parents

| CPM (frequency in past 3 months) | Mean ± SD | N (%) |
| --- | --- | --- |
|  |  |  |
| *Minor CPM* | **4.2± 6.6** | **254 (62.9%)** |
| Pinched, shook, pushed or shoved a child | 2.5**±**3.75 | 186 (46 %) |
| Hit child’s hand, back, arm or leg with hand | 2.5**±**3.9 | 187 (46.3 %) |
| Hit child’s buttocks with hand | 0.4**±**1.3 | 43 (10.6 %) |
| *Severe CPM* | **1.6 ± 4.2** | **133 (32.9%)** |
| Hit child’s buttocks with an object | 0.3**±**1.5 | 20 (5%) |
| Hit child’s face or head with hand | 0.7**±**1.5 | 85 (21%) |
| Kicked a child with a foot or hit with a fist | 0.3**±**1.4 | 39 (9.7%) |
| Hit elsewhere (not buttocks) with an object | 0.7**±**2.2 | 64 (15.8%) |

SD: standard deviation, CPM; Child physical maltreatment

Figures (S1). Curve estimation of significant predictors of minor and severe CPM frequency (in multiple regression)


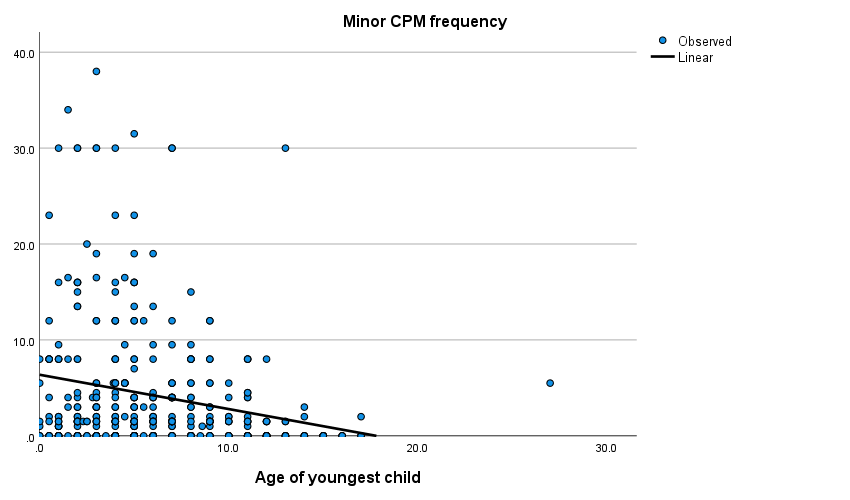


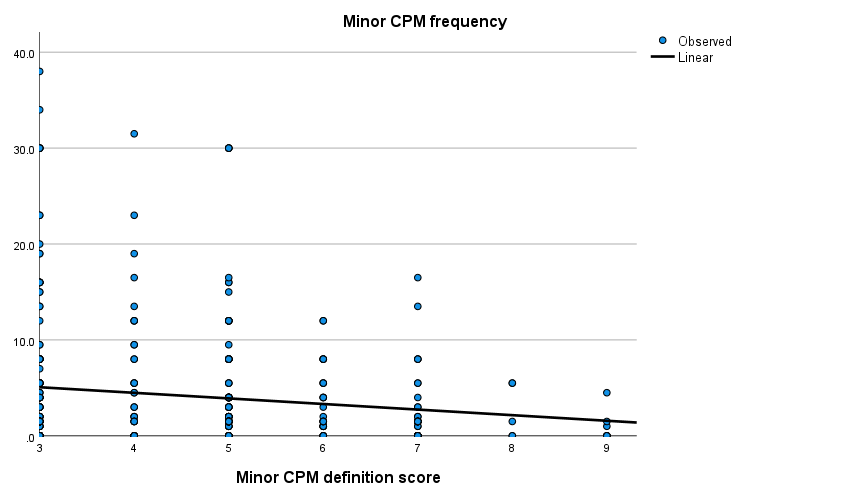


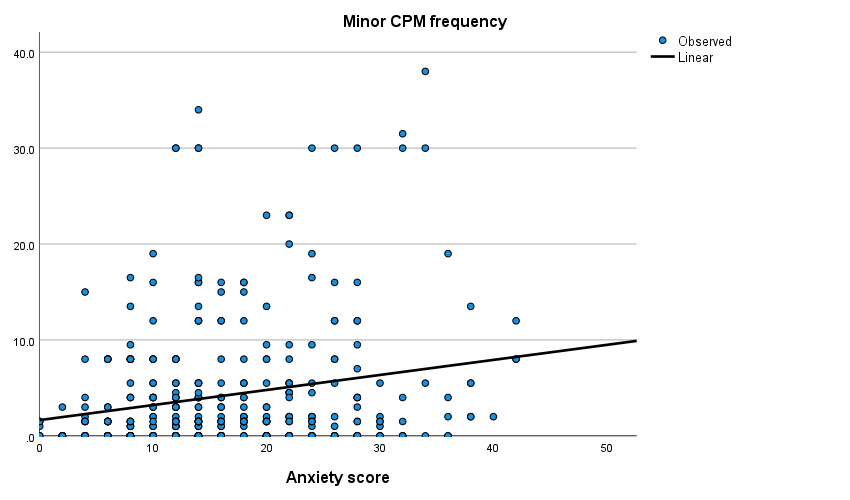


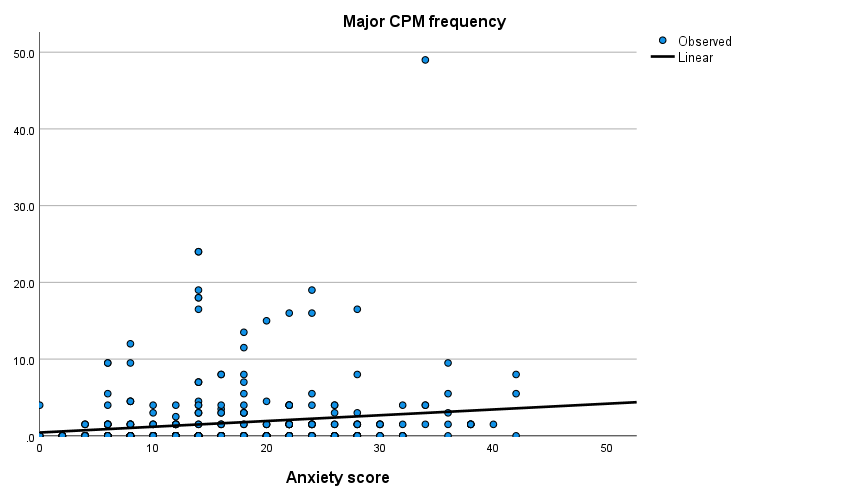


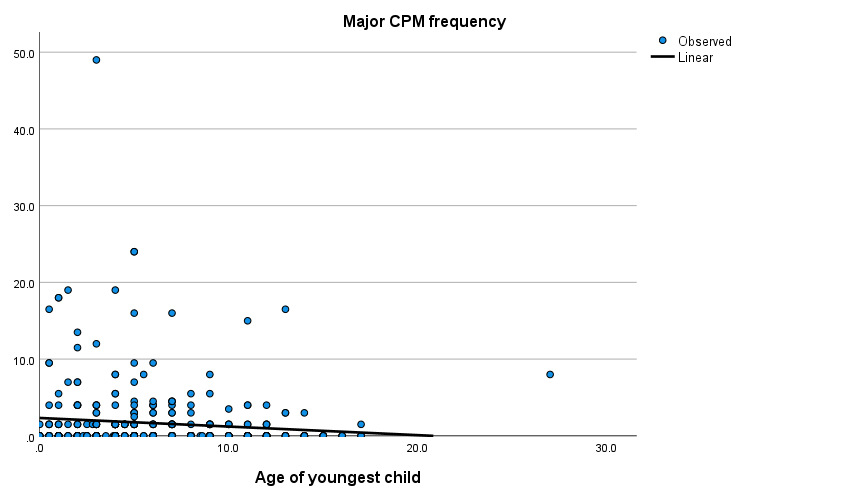


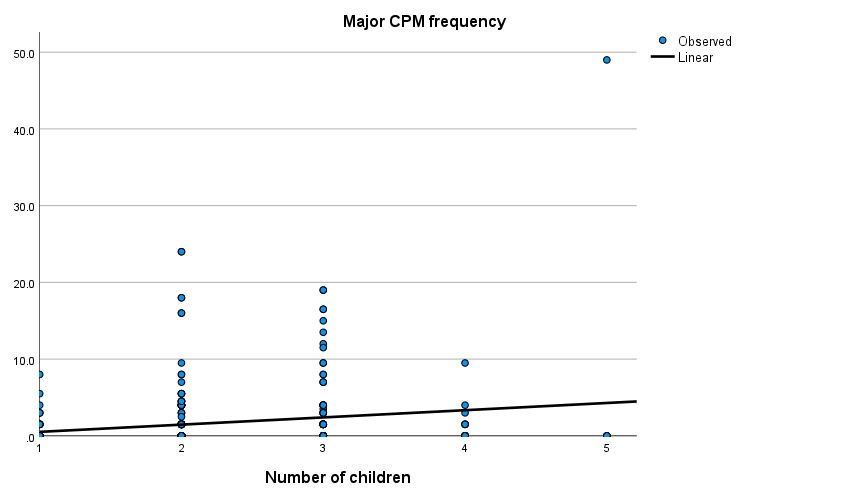


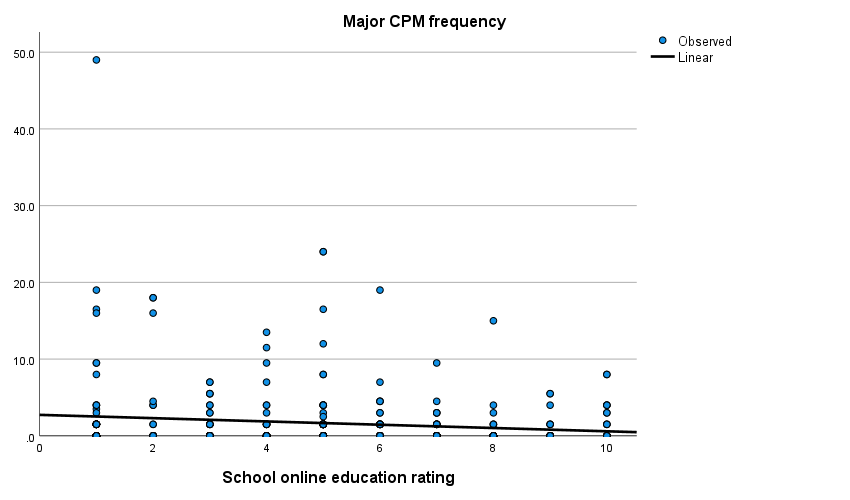

Supplement: Supplementary file 1 — Additional file 1: Table(S1). CPM 7-items frequency during past 3 months reported by the studied participating parents. Figures (S1). Curve estimation ofsignificant predictors of minor and severe CPM frequency (in multipleregression). [file 12888_2023_4947_MOESM1_ESM.docx]
